# Genetic variation of barley genotypes using morphological traits, amylose content, and molecular markers

**DOI:** 10.1038/s41598-025-19242-w

**Published:** 2025-10-07

**Authors:** Suraia Rahman Aka, Umakanta Sarker, Mohammad Zahirul Alam Talukder, Md. Golam Azam, Sezai Ercisli, Nazmul Hossain, Mohammed S. Alharbi, Riaz Ullah

**Affiliations:** 1 Department of Genetics and Plant Breeding, Faculty of Agriculture, Gazipur Agricultural University, Gazipur, 1706 Bangladesh; 2 Plant Breeding Division, Bangladesh Agricultural Research Institute, Gazipur, 1701 Bangladesh; 3 Pulses Research Centre, Bangladesh Agricultural Research Institute, Ishurdi, Pabna, 6620 Bangladesh; 4 Department of Horticulture, Faculty of Agriculture, Ataturk University, 25240 Erzurum, Turkey; 5 Department of Agronomy, Iowa State University, Ames, IA 50010 USA; 6 Department of Pharmacognosy, College of Pharmacy, King Saud University, 11451 Riyadh, Saudi Arabia

**Keywords:** Plant breeding, Agricultural genetics, Genetic markers

## Abstract

For any plant breeding endeavor to be effective, a variety of genetic resources must be available and accessible. Using simple sequence repeats (SSRs) markers, amylose content, and agro-morphological characterization, the genetic diversity of 50 barley genotypes was evaluated. Across 50 genotypes, analysis of variance revealed highly significant variation (*p* < 0.01) in each trait. The genotypes G20, G7, G18, G28, G41, G45, G50, G13, G39, and G47 displayed the highest yields. Positive correlations were found between the number of tillers per plant, plant height, spike length, number of grains per spike, 1000-grain weight, and grain yield. Cluster analysis sorted genotypes into five different groups. Cluster I had a minimum of four genotypes, and a maximum of forty-four genotypes were found in cluster V. Ninety-nine percent of the overall diversity among genotypes was accounted for by the first five main principal components (PCs). The contents of amylose and amylopectin were 13–29% and 71–87%, respectively. The genotypes G4, G5, G8, G12, G19, G34, G6, G37, and G41 exhibited the greatest amylose content. These genotypes might be chosen to increase quality and achieve the desired levels of amylose and amylopectin. Significant genetic variety was revealed among 50 barley genotypes by molecular analysis using 20 SSR markers. They had an average of 2.95 alleles per locus, ranging from 2 to 4 alleles. With an average of 0.52 per locus, the polymorphism information content (PIC) value varied from 0.31 to 0.67. A similarity matrix was created using the Unweighted Pair Group Method with Arithmetic Mean methodology, which divided the tested genotypes into five major groups for the determination of the genetic relatedness of barley genotypes. The SSR-based analysis clustered the genotypes into four different groups. The different grouping based on agro-morphological and SSR markers suggests a relationship between allelic profiling and expression profiling, or between genomic DNA and agro-morphological traits. It could be very helpful for future barley research. Barley genotype identification may benefit from the identified DNA markers. Similar to this, the high level of genetic diversity observed in the genotypes may be helpful in developing strategies for the management and conservation of barley germplasm as well as in the future for choosing parents from a variety of backgrounds for use in breeding programs.

**Supplementary Information:**

The online version contains supplementary material available at 10.1038/s41598-025-19242-w.

## Introduction

Barley (*Hordeum vulgare* L.) is one of the world’s most ancient food crops. Since the early phases of agricultural innovation 8000–10,000 years ago, it has been a major cereal crop^1^. It is a commercially significant cereal crop, ranking fourth in the world behind wheat, rice, and maize in terms of both the amount produced and the area cultivated^2^. The annual world harvest of barley in the late century was approximately 140 million tonnes from about 55 million ha^3^. It is a self-pollinating diploid crop^4^. Because of its higher adaptability, it is commonly produced as a summer crop in temperate locations and as a winter crop in tropical ones. It is a short-season rabi cereal that can withstand drought. Growing barley has several advantages, including erosion management, nutrient recycling, weed and insect control, soil restructuring, and building forage/legume stands. Compared to other cereal crops, it is the most widely adapted grain, growing in both fertile and arid environments. It is a major food source in Asia and northern Africa^5^. Barley is indeed a versatile grain used for both food and feed, and it plays a vital role in the production of malt, particularly for brewing beer^6^. While a significant portion of barley is used for animal feed, a specific type called “malting barley” is carefully cultivated to meet the quality standards required for malting and brewing. It is considered a food that is high in nutrients, low in calories, and less starchy than wheat and/or rice. The barley grain contains vitamins, minerals, lipids, proteins, and carbohydrates that are essential for healthy human development and growth. Dietary fiber, iron, copper, manganese, and selenium are also present. The main carbohydrate in barley is starch, ranging from 62 to 77% of its dry weight^7,8^. Amylose and amylopectin are the two components of starch. According to its amylose content, barley can be divided into three types: normal (25–27% amylose), waxy (non-detectable and below 5%), and high amylose type (> 35% amylose)^7,9^. Amylose has a linear chain of D-glucose with a low series of polymerization (104 units), whereas amylopectin exhibits several series of polymerization (105–106 units). The variation in amounts of amylose and amylopectin is responsible for its unique physical and chemical properties, with strong influences on the functional properties of flour or semolina and its specific uses in food^10,11^. Certain physicochemical characteristics of starch are critical to the product’s final application, such as its gelatinization; gelation and pasting are dependent on the amylose to amylopectin ratio. To increase the amount of amylose in barley, a deeper comprehension of the genetic process governing amylose metabolism is necessary. The amylose (*amo1*) and waxy (*wax*) genes, respectively, regulate the concentrations of amylose and amylopectin in barley. The single recessive gene *amo1* is responsible for the amylose content of up to 45%^12,13^. The *amo1* gene is located on chromosome 1H^11^, and the *wax* gene is located on chromosome 7H^14^. Different amounts of amylose are present in the grain as a result of the interaction between these two genes. Agro-morphological diversity is the first step in classifying and selecting crop germplasm^15^. It is the foundation of genetic diversity, and research at any taxonomic level^16^. It has been used to establish crop collections, identify duplicates, discriminate between materials from different geographic areas, and prioritize materials for use in breeding programs^17,18^.

Genetic diversity with molecular markers is an integral aspect of any agricultural production system in breeding, modification in germplasm effectively and recognizing the importance of genetic variation in the germplasm of crop organisms^19^. It has been demonstrated that molecular markers are useful instruments for characterizing and assessing genetic diversity both within and between species and populations. By introducing novel and advantageous features from related grass species and land races, these markers have been used to monitor variations in DNA sequences within and across the species, as well as to generate new sources of genetic diversity. Different molecular markers like ISSR, CDDP, and SCoT can be used to assess genetic variation and population structure in barley genotypes^20^. These markers, based on DNA differences, allow researchers to study the diversity and relationships among different barley varieties or populations. SSR or microsatellite markers have recently been created for important crop plants, and it is anticipated that this marker system would accelerate the creation of new markers and their application in breeding initiatives. Microsatellites have been successfully applied for the detection of genetic diversity^21^, genome mapping^22,23^, marker-assisted selection of agronomically important traits^24^ and genotype differentiation^25,26^. The Bangladesh Agricultural Research Institute’s (BARI) Plant Breeding Division possesses a sizable collection of barley lines and cultivars. However, starch quality content (Amylopectin and amylose) and its variation, as well as genetic variation at agro-morphological and molecular level of the barley genotypes, have not yet been analyzed. Keeping this in view, the following objectives have been planned:

1. To examine the molecular marker and agro-morphological trait-based divergence of barley genotypes.
2. To determine the utmost exceptional barley genotypes for amylose and amylopectin for future research, as well as to approximate the variance in starch characteristics in barley.

## Materials and methods

### Evaluation of genetic diversity in barley germplasm using agromorphological characteristics

#### Plant material

50 barley genotypes were evaluated in this study. These genotypes were obtained from the Bangladesh Agricultural Research Institute (BARI), Gazipur. BARI received these barley genotypes from ICARDA due to collaboration with this international institution. Detailed information on genotypes is given in Table 1. The seeds of the barley genotypes used for the study comply with our institutional, national, and international guidelines and legislation.

**Table 1:** Barley genotypes evaluated in the study.

Serial no	Genotypes with pedigree	Code	Serial no	Genotypes with pedigree	Code
1	IBON-22/E80	G1	26	IBON-22/E10	G26
2	IBON-22/E51	G2	27	IBON-22/E68	G27
3	IBON-22/E103	G3	28	IBON-22/E66	G28
4	IBON-22/E58	G4	29	IBON-22/E79	G29
5	IBON-22/E67	G5	30	IBON-22/E36	G30
6	IBON-22/E38	G6	31	IBON-22/E69	G31
7	IBON-22/E75	G7	32	IBON-22/E4	G32
8	IBON-22/E65	G8	33	IBON-22/E55	G33
9	IBON-22/E57	G9	34	IBON-22/E56	G34
10	IBON-22/E63	G10	35	IBON-22/E18	G35
11	IBON-22/E9	G11	36	IBON-22/E7	G36
12	IBON-22/E16	G12	37	IBON-22/E41	G37
13	IBON-22/E49	G13	38	IBON-22/E20	G38
14	IBON-22/E93	G14	39	IBON-22/E50	G39
15	IBON-22/E88	G15	40	IBON-22/E101	G40
16	IBON-22/E5	G16	41	IBON-22/E33	G41
17	IBON-22/E53	G17	42	IBON-22/E12	G42
18	IBON-22/E3	G18	43	IBON-22/E28	G43
19	IBON-22/E40	G19	44	IBON-22/E39	G44
20	IBON-22/E89	G20	45	IBON-22/E45	G45
21	IBON-22/E1	G21	46	IBON-22/E19	G46
22	IBON-22/E34	G22	47	IBON-22/E60	G47
23	IBON-22/E22	G23	48	IBON-22/E76	G48
24	IBON-22/E2	G24	49	IBON-22/E103	G49
25	IBON-22/E74	G25	50	IBON-22/E14	G50

#### Experimental designs and trial management

At the BARI research field in Gazipur, fifty barley genotypes were assessed in the rabi season of 2021–2022. RCBD design was followed for sowing the seeds. Three replications of each genotype were sown in two rows of five meters each, with a row-to-row spacing of twenty-five centimeters. To grow the crop, fertilizers (N, P, and K) were administered at a rate of 100:60:40 kg/ha. The experiment was designed with the greatest possible care to reduce any confounding variables that might have an impact on the study’s anticipated outcome. Every other management technique, such as hand weeding (three times throughout the seedling and vegetative stages), was used consistently. To compute the data, five randomly selected plants from each genotype were used. Statistical analysis was performed on the data using the Crop Stat 7.2 program.

### Agro-morphological traits

Using the following methods, five plants were chosen at random to record the agro-morphological trait data:

#### Days to 50% heading

Counting began on the day of sowing and continued until 50% of the heads had fully blossomed.

#### Days to 75% maturity

The number of days from sowing to the point at which 75% of a plot reached maturity was known as the "days to maturity."

#### Plant height (cm)

Plant height (cm) was calculated as the average of 10 randomly selected plants in each plot, and it was stated as the height in centimeters from the soil surface to the tip of the spike (excluding the awn at maturity).

#### Spike length (cm)

The average of 10 randomly selected plants was used to calculate the main plant’s spike length, which was measured in centimeters from base to tip, omitting the awns.

#### Number of tillers per plant

An average of 10 randomly selected plants from each plot were used to calculate the number of tillers (spike-bearing plants) for each plant.

#### Number of seeds per spike

The quantity of seeds produced by each plant’s primary tiller was counted, and the result was an average of ten randomly chosen plants from each plot.

#### Thousand seed weight (g)

The thousand seed weight was calculated by weighing one thousand randomly chosen seeds from each plot and controlling their moisture to 12.5%.

#### Grain yield (t ha^−1^)

Grain yield was measured and expressed in t ha^−1^ after the grain from each plot was adjusted to have a moisture level of 12.5%.

### Estimation of amylose and amylopectin

#### Flour sample preparation

Following grinding, grain samples were processed using the JFS-13A milling machine from Tekpa Laboratory (which has a 0.5 mm sieve). In between each sample, the mill was cleaned.

#### Amylose and amylopectin measurement methods

To determine the apparent amylose and amylopectin ratio, over 100 mg was utilized. For standard testing, the iodine-potassium iodide (I: KI) procedure was modified^27^. The earliest reports of iodine-potassium iodide staining were for measurements of potato amylose^28^. Every test was conducted at least twice. Using a Shimadzu UV-1800 Spectrophotometer (China), absorbance was measured at 620 and 443 nm for amylose and 525 and 725 nm for amylopectin to estimate their respective contents.

#### Statistical analysis

To ascertain the degree of genetic diversity among the barley genotypes, the mean of the data gathered from agro-morphological variables was computed and examined using basic statistical techniques such as mean, minimum, maximum, standard deviation, and coefficient of variation (CV). The method of Steele and Torrie^29^ was followed to establish simple correlation coefficients between all pairs of quantitative features using mean values. According to Sneath and Sokal^30^, the data of the entire set of agro-morphological features was examined using numerical taxonomic procedures using principal component analysis (PCA) and cluster analysis. Before doing the cluster analysis and PCA, the means of each attribute were normalized using Z-scores in order to eliminate scaling differences. For every pair of genotypes, estimates of the Euclidean distance coefficients were performed.

### Assessment of genetic diversity using microsatellite (SSR) markers in barley germplasm

#### Plant material

To estimate the level of genetic diversity among 50 genotypes of barley, microsatellites or SSR marker were used.

#### Extraction of DNA

DNA was extracted from young leaves by using CTAB method^31^. Qualification and quantification of extracted DNAs were determined by 0.8% agarose gel electrophoresis and spectrophotometer at 260 nm, respectively^32^.

#### Specification of the primers

After reviewing the literature on the valuation of genetic diversity in barley, a variety of SSR markers were chosen for the molecular characterization trial (Table 2). A further twenty (20) SSR primers encompassing the entire barley genome were employed to clarify the genetic diversity estimate.

**Table 2:** Primer details used in the trial.

	Primer name	Sequence	References	Chr
1	Bmag0211	F: ATTCATCGATCTTGTATTAGTCC	Ali et al.^33^	1H
		R: ACATCATGTCGATCAAAGC		
2	GBM1411	F: TGCTCTACAAACCGCTGATG	Gougerdchi et al*.*^34^	1H
		R: CGGAGTTCAGTTCAGACCGT		
3	Bmac0213	F: ATGGATGCAA GACCAAAC	Elakhdar et al.^35^	1H
		R: CTATGAGAGGTAGAGCAGCC		
4	GBM1468	F: GTTCCTTCTTTGCCTCCTCC	Gougerdchi et al.^34^	2H
		R: TCCATGGTCCTCTCCTTCAC		
5	Bmac0134	F: CCAACTGAGTCGATCTCG	Ali et al.^33^	2H
		R: CTTCGTTGCTTCTCTACCTT		
6	Bmag0749	F: CGGATTCTTGAGTAGTCTCTG	Elakhdar et al.^35^	2H
		R: GATCTGTTTTTGTAGAACATGC		
7	GBM1280	F: CTTCTTCTTCTTGTTGGGCG	Gougerdchi et al.^34^	3H
		R: AAGGGATCAGTTTGGTTCCC		
8	scssr25691	F: ACGAGCTGATATCCCACGAG	Gougerdchi et al.^34^	3H
		R: TCCGAGCTTCTTATCTTTGG		
9	Bmag0023	F: AACACAGACCTACGGGTC	Wang et al.^36^	3H
		R: CATGAGATAGATCCAAGCAC		
10	scssr20569	F: ATCGAGCACCTACGAACC	Gougerdchi et al.^34^	4H
		R: TTGCATAGCGGAAGTAATCC		
11	HVLEU	F: TTGGAAGTGTACAGCAATGGAG	Misganaw et al.^37^	5H
		R: TGAAAGGCCCCACAAGATAG		
12	Bmag0375	F: CCCTAGCCTTCCTTGAAG	Misganaw et al.^37^	4H
		R: TTACTCAGCAATGGCACTAG		
13	HvLOX	F: CAGCATATCCATCTGATCTG	Ferreira et al.^38^	5H
		R: ACACCCTTATTTATTGCCTTAA		
14	Bmac0163	F: TTTCCAACAG AGGGTATTTA CG	Polgari et al.^39^	5H
		R: GCAAAGCCCATGATACATACA		
15	HVM65	F: AGACATCCAAAAAATGAACCA	Ferreira et al.^38^	6H
		R: TGGTAACTTGTCCCCCAAAG		
16	Bmac0018	F: GTCCTTTACGCATGAACCGT	Gougerdchi et al.^34^	6H
		R: ACATACGCCAGACTCGTGTG		
17	Bmac0040	F: AGCCCGATCAGATTTACG	Ali et al*.*^33^	6H
		R: TTCTCCCTTTGGTCCTTG		
18	Bmag0135	F: ACGAAAGAGTTACAACGGATA	Backes et al.^40^	7H
		R: GTTTACCACAGATCTACAGGTG		
19	GBM1102	F: GGCATCTGCTCTTGATGACA	Gougerdchi et al.^34^	7H
		R: GGAGGAAAATAGGCACGGAT		
20	EBmac0603	F: ACCGAAACTA AATGAACTAC TTCG	Ali et al.^33^	7H
		R: TGCAAACTGTGCTATTAAGGG		

Chr = Chromosome, F = Forward, R = Reverse.

#### A polymerase chain reaction analysis with simple sequence repetitions

For microsatellite analysis of barley genotypes, the polymerase chain reaction protocol was modified by carefully regulating the ratios of different reagents and genomic DNA in the master mixture for PCR. The outcomes were recorded using agarose gels with varying percentages. In a similar vein, modifications were made to the temperature and duration of different polymerase chain reaction steps and their impact on PCR amplification to potentially obtain high-quality products for genetic characterization. The optimal PCR reaction mixture had a volume of 20 μL and contained the following ingredients: 12 μL of ddH_2_O, 10 mM Tris–HCl pH 8.3, 50 mM KCl, MgCl_2_ (1.5 mM), 200 μM of dNTPs mixture, 0.4 μM of primer forward and primer reverse, 1 unit of Taq polymerase, and about 20 ng of template DNA. Utilizing a GenePro model TC-G-96E heat cycler (M/s. Bioer, Hangzhou Bioer Technology Co. Ltd.), the subsequent PCR conditions were used for every PCR: 45 s of primer annealing at 60 °C, 1 min of primer extension at 72 °C, and 5 min of final extension at 72 °C. The first denaturation was carried out at 94 °C for 5 min. For every PCR reaction, three technical duplicates were run. After the 2.0% agarose gel was used to verify that the PCR amplicon produced the desired result, the remaining PCR product was prepared for sequencing from M/s. PVT. Sequencher Ltd.

#### Electrophoresis of amplified products

After amplification, 3 μL of loading dye (bromophenol blue = 0.4%, xylene cyanole FF = 0.4%), glycerol = 5 mL, and 20 μL of the generated products were added to each PCR tube. Ten (10) μL of the products were loaded at a 3% concentration in 1 × TBE buffer (Tris–Borate = 10 mM and EDTA = 1 mM) onto Gene Choice High-Resolution agarose (CLP, USA) gels. During the gel preparation process, 0.7 μg mL^-1^ of ethidium bromide was previously combined. Invitrogen, Life Technologies) provided a 50 bp DNA ladder that was utilized as a marker to determine the molecular weight of the amplified SSR products. After electrophoresis, images of the agarose gels were taken with the UVI Doc Gel Documentation System (Alpha Innotech Corp., Suit – 100, USA) using the software Alpha imager HP.

#### Allele scoring and data analysis

To score the data for the study of SSR markers, images of the agarose gels stained with ethidium bromide were utilized. Using the relative primer pair, each band was regarded as an amplified DNA fragment and counted as a unit characteristic. These bands were given a score of 1 for the presence of the DNA fragment and 0 for its absence. Band number one was scored from the top to the bottom. The genotypes were numbered from left to right, starting with lane number one, except for the lanes on the far left and far right, which were loaded with a DNA ladder of known molecular weight as a marker for estimating the molecular size of the amplified product. A binary matrix was created using the data that was gathered depending on whether the bands were present or absent. The majority of variant primers were found by considering the level of polymorphism. Each primer pair’s polymorphic information content (PIC) value was determined using the Anderson et al*.*^41^ methodology. The total number of alleles, number of polymorphic alleles, difference between the smallest amplified allele size and the biggest one at any SSR locus, and PIC value were all calculated using the same methodology for each primer pair based on barley genotypes.

## Results

Fifty different barley genotypes were examined in this study. To determine the degree of genetic diversity at the phenotypic level, the genetic characterization was conducted in the open field using agro-morphological markers, amylose content, and SSR markers to obtain a comprehensive and trustworthy representation of potential genetic variability at the DNA level.

### Genetic diversity assessment in barley genotypes based on agro-morphological traits and amylose content

Nine quantitative parameters were utilized to define each genotype to provide insight into the genetic diversity seen in the barley germplasm. Table 3 provides the fundamental statistical information (mean, minimum, maximum, standard deviation, CV, and variance) for each quantitative attribute identified for each genotype. For a variety of quantitative variables, the genotypes’ patterns of variability varied. Variations of significance were noted for every attribute across all barley genotypes. Table 3 displays the mean performance of the agromorphological parameters and the amylose content.

**Table 3:** Basic statistical analysis of agro-morphological traits and amylose content of barley.

Traits	Mean	Minimum	Maximum	SD	CV (%)	Variance	F-test
Days to 50% heading	66.6	62.0	74.0	4.3	6.3	18.4	**
Days to 75% maturity	108.0	105.0	112.0	3.0	2.9	8.9	**
Plant height (cm)	74.8	67.2	90.4	6.5	5.3	42.4	**
Number of tillers per plant	6.0	4.8	7.6	0.9	9.9	0.7	**
Spike length (cm)	7.0	5.0	9.2	1.2	9.5	1.4	**
Number of grains per spike	34.3	24.8	50.8	8.1	8.2	65.2	**
1000-grain weight (g)	37.2	28.0	50.0	5.8	8.0	34.0	**
Grain yield (t ha^-1^)	3.3	2.5	4.1	0.5	6.8	0.2	**
Amylose content (%)	23.9	13	29	3.4	4.8	11.6	**

SD = standard deviation, CV = coefficient of variation, ** indicates significant at 1% level of significance.

#### Days to 50% heading

The number of days to 50% heading showed a broad range of variation. With a mean value of 67 days and a range of 62–74 days, the coefficient of variance was 6.3% (Table 3). With 74 days to heading, genotypes G17 and G26 were the most longest, and genotypes G2, G27, and G49 were the earliest, with 62 days to 50% heading (Table 4).

**Table 4:** Mean performance of 50 different barley genotypes based on agro-morphological traits and amylose content.

Sl. No	Entry	DH	DM	PH (cm)	NTP	SL	NGP	TGW	GY	AL
1	G-1	64.0	107.0	71.2	5.2	7.8	35.6	40.0	3.8	24.0
2	G-2	62.0	105.0	70.4	4.8	7.0	47.0	35.0	3.5	22.0
3	G-3	67.0	108.0	71.6	4.8	6.6	28.0	34.0	3.0	21.0
4	G-4	68.0	107.0	68.2	6.2	5.6	30.2	45.0	3.4	29.0
5	G-5	63.0	105.0	72.0	5.0	6.4	27.6	33.0	2.9	28.0
6	G-6	67.0	110.0	74.6	6.8	7.8	40.4	45.0	3.6	27.0
7	G-7	69.0	112.0	77.4	7.2	7.2	48.8	50.0	4.0	26.0
8	G-8	68.0	110.0	73.0	5.0	7.2	33.8	35.0	3.2	28.0
9	G-9	66.0	107.0	72.8	5.0	5.4	27.2	35.0	2.5	25.0
10	G-10	63.0	105.0	69.8	6.6	6.8	33.4	40.0	3.5	22.0
11	G-11	67.0	107.0	72.8	6.2	6.6	29.6	33.0	3.1	23.0
12	G-12	68.0	109.0	78.8	6.0	6.6	33.4	34.0	3.2	28.0
13	G-13	68.0	109.0	90.2	7.4	9.0	44.0	35.0	3.9	24.0
14	G-14	65.0	108.0	78.2	4.8	5.0	32.4	34.0	2.7	19.0
15	G-15	70.0	111.0	85.8	7.6	8.2	50.0	40.0	3.8	23.0
16	G-16	72.0	112.0	90.4	4.8	7.4	40.8	35.0	3.8	26.0
17	G-17	74.0	112.0	70.0	4.8	7.0	26.0	35.0	3.0	25.0
18	G-18	70.0	110.0	81.2	6.0	7.2	26.0	45.0	4.0	22.0
19	G-19	69.0	107.0	69.4	5.0	5.6	25.0	30.0	2.8	28.0
20	G-20	69.0	108.0	79.0	6.6	7.6	44.0	46.0	4.1	24.0
21	G-21	64.0	106.0	74.4	6.0	7.2	27.2	30.0	2.8	20.0
22	G-22	73.0	111.0	70.2	6.2	7.4	35.2	38.0	3.7	19.0
23	G-23	63.0	105.0	67.8	5.4	8.6	33.4	40.0	3.3	23.0
24	G-24	64.0	107.0	71.8	5.6	6.2	27.6	30.0	3.0	26.0
25	G-25	63.0	105.0	75.4	5.6	6.7	24.8	35.0	3.1	25.0
26	G-26	74.0	111.0	73.0	6.0	7.8	28.1	35.0	3.0	24.0
27	G-27	62.0	105.0	82.0	5.9	7.2	28.4	35.0	3.4	25.0
28	G-28	70.0	109.0	81.2	7.2	9.2	40.4	48.0	4.0	26.0
29	G-29	64.0	106.0	71.4	5.6	5.2	25.6	30.0	2.8	23.0
30	G-30	65.0	106.0	71.4	5.6	6.5	30.6	35.0	3.3	27.0
31	G-31	68.0	109.0	76.0	5.4	5.4	40.4	45.0	3.4	26.0
32	G-32	71.0	110.0	74.2	5.4	5.8	28.2	30.0	2.6	20.0
33	G-33	64.0	107.0	70.8	6.2	7.0	50.8	35.0	3.6	18.0
34	G-34	68.0	109.0	69.2	6.4	7.4	45.2	40.0	3.7	28.0
35	G-35	68.0	109.0	68.0	7.0	8.6	34.8	45.0	3.8	23.0
36	G-36	64.0	108.0	67.2	5.6	5.2	25.2	33.0	2.6	27.0
37	G-37	65.0	109.0	69.2	6.2	6.2	28.2	33.0	3.0	27.0
38	G-38	66.0	108.0	71.2	6.6	6.6	39.0	47.0	3.8	25.0
39	G-39	66.0	107.0	75.4	6.6	8.0	36.0	45.0	3.9	15.0
40	G-40	65.0	106.0	73.0	6.4	7.2	29.2	35.0	2.9	24.0
41	G-41	63.0	106.0	74.6	6.6	9.2	44.4	40.0	4.0	27.0
42	G-42	64.0	107.0	75.4	5.4	5.4	26.4	28.0	2.7	26.0
43	G-43	68.0	110.0	70.0	5.6	6.8	29.2	35.0	2.8	13.0
44	G-44	65.0	107.0	70.2	5.6	6.0	32.8	38.0	3.2	23.0
45	G-45	68.0	110.0	84.8	6.6	7.6	50.0	40.0	4.0	24.0
46	G-46	67.0	108.0	76.0	5.8	6.0	27.6	30.0	2.7	25.0
47	G-47	67.0	108.0	76.0	6.8	9.0	32.6	35.0	3.9	23.0
48	G-48	65.0	109.0	89.8	6.6	6.8	33.6	36.0	3.2	24.0
49	G-49	62.0	105.0	71.6	6.2	6.2	27.8	33.0	3.1	20.0
50	G-50	64.0	107.0	81.8	7.1	8.2	48.0	40.0	4.0	24.0
Mean		67.0	108	74.8	6.0	7.0	34.3	37.2	3.3	23.9

DH, Days to 50% heading; DM, Days to 75% maturity; PH, Plant height (cm); NTP, Number of tillers per plant; SL, Spike length (cm); NGP, Number of grains per spike; TGW: 1000-grain weight (g); GY, Grain yield (t ha^-1^); AL, Amylose content.

#### Days to 75% maturity

The range of variance was seen in the days to 75% maturity. Its mean value was 108 days, and its range was 105–112 days. Its coefficient of variance was 2.9% (Table 3). Table 4 shows that the genotypes G7, G16, and G17 had the longest maturation times at 112 days, whereas the genotypes G2, G10, G23, G25, G27, and G49 had the earliest maturation times at 105 days.

#### Plant height (cm)

Plant height showed a significant degree of heterogeneity across all genotypes evaluated. During a field trial, it ranged from 67.2 to 90.4 cm with a mean value of 74.8 cm and a CV of 5.3% (Table 3). According to Table 4, the tallest and shortest genotypes were G16 and G36, respectively.

#### Number of tillers per plant

The current genotypes showed variance in the number of tillers per plant ranging from 4.8 to 7.6, with a mean value of 6.0 and a CV of 9.9% (Table 3). According to Table 4, the genotypes G2, G3, G14, G16, and G17 had the fewest tillers per plant (4.8), whereas the genotype G15 plant had the most tillers (7.6).

#### Spike length (cm)

With a mean value of 7.0 cm and a CV of 9.5%, the spike length showed a considerable degree of variability (Table 3). It was found that the genotypes G14 produced the shortest spikes, while the genotypes G28 and G41 produced the longest spikes (Table 4).

#### Number of grains per spike

With a mean value of 34.3 grains per spike and a CV of 8.2%, the tested genotypes displayed a high degree of variation in the number of grains per spike, which ranged from 24.8 to 50.8 (Table 3). G23 genotype had the highest grains per spike, while G25 genotype (Table 4) had the lowest oner.

#### 1000 grains weight (g)

Weight in 1000 grains is regarded as one of the key quantitative characteristics. A 1000-grain weight adjustment was made at a moisture level of 12%. The genotypes under investigation exhibited a significant degree of variance, ranging from 28 to 50 g, with a mean value of 37.2 g and an 8.0% CV (Table 3). G7 grains had the highest weight, whereas G42 grains had the lowest weight (Table 4).

#### Grain yield (t ha^−1^)

Agromorphological parameters of great importance include grain yield (t ha^−1^). The grain yield (t ha^−1^), with a mean value of 3.3 t ha^−1^ and a CV of 6.8%, showed a remarkable degree of variance, ranging from 2.5 to 4.1 t ha^−1^ (Table 3). Table 4 shows that genotypes G20 (4.1 t ha^−1^) had the highest grain yield, followed by G7 (4.0 t ha^−1^), G18 (4.0 t ha^−1^), G28 (4.0 t ha^−1^), G41 (4.0 t ha^−1^), G45 (4.0 t ha^−1^), G50 (4.0 t ha^−1^), G13 (3.9 t ha^−1^), G39 (3.9 t ha^−1^) and G47 (3.9 t ha^−1^); genotype G9 (2.5 t ha^−1^) had the lowest grain yield, followed by G26 (2.6 t ha^−1^) and G42 (2.7 t ha^−1^).

#### Amylose content (%)

Tables 4 and 5 display the amylose and amylopectin content of the barley genotypes. Table 3 indicates that there was a significant difference (CV, 4.8%) in the apparent amylose concentration among barley genotypes. The range of amylose content of barley genotypes was 13.0–29.0% with a mean value of 23.9% whereas the amylopectin content of barley genotypes ranged between 71.0 and 87.0% with a mean value of 76.1%. G4 produced the maximum amount of apparent amylose content (29.0%) with 71% of amylopectin among the barley genotypes, while G43 produced the lowest amount (13.0%) with 87.0% of amylopectin (Table 5). The top ten genotypes for producing amylose were G4 (29.0%), G5 (28.0%), G8 (28.0%), G12 (28.0%), G19 (28.0%), G34 (28.0%), G6 (27.0%), G37 (27.0%), and G41 (27.0%). In terms of amylopectin content, the nine genotypes with the highest levels of amylopectin content were G43 (87.0%), G39 (85.0%), G33 (82.0%), G14 (81.0%), G22 (81.0%), G21 (80.0%), G32 (80.0%), G49 (80.0%), and G3 (79.0%).

**Table 5:** Amylose and amylopectin content in barley genotype.

Serial no	Genotypes	Amylose (%)	Amylopectin (%)	Serial no	Genotypes	Amylose (%)	Amylopectin (%)
1	G1	24.0	76.0	26	G26	24.0	76.0
2	G2	22.0	78.0	27	G27	25.0	75.0
3	G3	21.0	79.0	28	G28	26.0	74.0
4	G4	29.0	71.0	29	G29	23.0	77.0
5	G5	28.0	72.0	30	G30	27.0	73.0
6	G6	27.0	73.0	31	G31	26.0	74.0
7	G7	26.0	74.0	32	G32	20.0	80.0
8	G8	28.0	72.0	33	G33	18.0	82.0
9	G9	25.0	75.0	34	G34	28.0	72.0
10	G10	22.0	78.0	35	G35	23.0	77.0
11	G11	23.0	77.0	36	G36	27.0	73.0
12	G12	28.0	72.0	37	G37	27.0	73.0
13	G13	24.0	76.0	38	G38	25.0	75.0
14	G14	19.0	81.0	39	G39	15.0	85.0
15	G15	23.0	77.0	40	G40	24.0	76.0
16	G16	26.0	74.0	41	G41	27.0	73.0
17	G17	25.0	75.0	42	G42	26.0	74.0
18	G18	22.0	78.0	43	G43	13.0	87.0
19	G19	28.0	72.0	44	G44	23.0	77.0
20	G20	24.0	76.0	45	G45	24.0	76.0
21	G21	20.0	80.0	46	G46	25.0	75.0
22	G22	19.0	81.0	47	G47	23.0	77.0
23	G23	23.0	77.0	48	G48	24.0	76.0
24	G24	26.0	74.0	49	G49	20.0	80.0
25	G25	25.0	75.0	50	G50	24.0	76.0
	CD (5%)					1.34	2.23
Mean						23.9	76.1
Range						13–29	71–87
Maximum						29	87
Minimum						13	71

#### Correlation studies

The simple correlation coefficient shown in Table 6 was explained by analyzing the mean of nine agro-morphological variables and the amylose content in fifty genotypes of barley. There was a link between these agro-morphological and starch-quality characteristics that was both favorable and negative. However, measurements of some functionally linked characters showed a substantial correlation with each other among the combinations of agro-morphological characters and amylose concentration during the agro-morphological investigation of barley. These traits included the plant’s morphology (such as plant height), traits related to flowering (such as days to 50% heading and days to 75% maturity), traits related to yield (such as number of tillers per plant, spike length (cm), number of grains per spike, 1000-grain weight (g), grain yield (t ha^-1^), and starch quality (such as amylose content). Days to 75% maturity and days to 50% heading showed a fairly strong positive connection (r = 0.86**). Plant height and days to 75% maturity also showed a strong positive correlation (r = 0.33*). Numerous variables, including the number of tillers per plant (r = 0.35*), spike length (r = 0.33**), number of grains per spike (r = 0.39**), and grain yield (r = 0.39**), showed a substantial positive connection with plant height. Numerous characteristics, including spike length (r = 0.59**), number of grains per spike (r = 0.52**), 1000-grain weight (r = 0.53**), and grain yield (r = 0.63**), exhibited a highly significant connection with the number of tillers per plant. The length of the spikes exhibited a highly significant positive association with the 1000-grain weight (r = 0.45**), grain yield (r = 0.74**), and number of grains per spike (r = 0.52**). There was a highly significant positive connection (r = 0.53**), (r = 0.45**), and (r = 0.55**) between 1000-grain weight and the number of tillers per plant, spike length, and number of grains per spike. Grains per plant (r = 0.76**), number of tillers per plant (r = 0.63**), spike length (r = 0.74**), number of grains per spike (r = 0.76**), and 1000-grain weight (r = 0.76**) were found to be positively correlated with the most important trait, grain yield (t ha^−1^).

**Table 6:** Correlation among agro-morphological traits and amylose content of barley.

Traits	DH	DM	PH	NTP	SL	NGP	TGW	GY	AL
DH	1.00								
DM	0.86**	1.00							
PH	0.20	0.33*	1.00						
NTP	0.08	0.17	0.35*	1.00					
SL	0.17	0.17	0.33*	0.59**	1.00				
NGP	0.09	0.26	0.39**	0.52**	0.52**	1.00			
TGW	0.22	0.26	0.12	0.53**	0.45**	0.55**	1.00		
GY	0.17	0.23	0.39**	0.63**	0.74**	0.76**	0.76**	1.0	
AL	0.02	0.02	0.04	-0.03	-0.08	-0.01	0.05	0.02	1.00

DH, Days to 50% heading; DM, Days to 75% maturity; PH, Plant height (cm); NTP, Number of tillers per plant; SL, Spike length (cm); NGP, Number of grains per spike; TGW: 1000-grain weight (g); GY, Grain yield (t ha^-1^); AL, amylose content. * and ** significant at 5% and 1% level of significance, respectively.

### Multivariate analysis of barley based on agro-morphological traits and amylose content

Two complimentary methods were used for the multivariate analyses of the agromorphological features and amylose content: PCA and cluster analysis. To show and assess the degree of phenotypic similarity and determine the relatedness among barley genotypes, the data are presented in the form of a dendrogram.

#### Cluster analysis

Based on nine agro-morphological parameters, a total of fifty barley genotypes were divided into five main clusters (I, II, III, IV, and V) via cluster analysis. As a result, Table 7 displays the five clusters that were produced, and Table 8 displays the genotype cluster mean. Due to the following characteristics: low plant height (71.5), the lowest number of tillers per plant (5.6), medium spike length (7.0), lowest number of grains per spike (29.3), low 1000-grain weight (34.6), low grain yield (3.0), and low amylose content (20.2), Cluster–I consisted of five genotypes that were grouped apart from other genotypes. Cluster II was made up of 14 genotypes that were distinguished from the other clusters by the following characteristics: low plant height (71.2), high 1000-grain weight (g) (Tables 7, 8), medium grain yield (3.6), medium amylose content (23.7), medium number of tillers per plant (6.1), medium spike length (7.3), medium number of grains per spike (38.8), and medium number of days to 50% heading (64.4) and maturity (107.2). Medium days to 50% heading (66.2), medium days to 50% maturity (1082.2), tall plant height (81.0), medium number of tillers per plant (6.0), medium spike length (7.0), medium number of grains per spike (31.1), medium 1000-grain weight (36.5), medium grain yield (3.4), and medium amylose content (23.5) were the distinguishing characteristics of Cluster-III, which comprised 6 genotypes. A total of eight genotypes made up Cluster-IV were distinguished from other genotypes by the presence of distinctive traits like the tallest plant height (83.8), the most tillers per plant (6.8), the maximum spike length (8.1), the most grains per spike (45.6), the maximum weight of 1000 grains (41.8), the highest grain yield (4.0), the medium number of days to 50% heading (68.8), the medium number of days to 75% maturity (109.8), and the high amount of amylose content (24.6) (Tables 7, 8). With the fewest days to 50% heading (65.1), the fewest days to 75% maturity (106.7), dwarf plant height (72.3), the fewest tillers per plant (6.0), the shortest spike length (6.2), the fewest grains per spike (27.2), the lowest 1000-grain weight (32.5), the lowest grain yield (2.9), and the highest amylose content (24.9), Cluster-V was made up of the maximum 17 genotypes and was isolated from other genotypes (Tables 7, 8).

**Table 7:** Total number of genotypes per cluster and percentage of barley genotypes.

Cluster	Total genotypes	Name of the genotypes	Percentage (%)
I	5	G17, G22, G26, G32, and G43	10
II	14	G1, G2, G4, G6, G10, G23, G31, G33, G34, G35, G38, G39, G41, and G44	28
III	6	G12, G14, G18, G27, G47, and G48	12
IV	8	G7, G13, G15, G16, G20, G28, G45, and G50	16
V	17	G3, G5, G8, G9, G11, G19, G21, G24, G25, G29, G30, G36, G37, G40, G42, G46, and G49	34

**Table 8:** The Mean values of clusters based on agro-morphological traits and amylose content.

Traits	Cluster- I	Cluster -II	Cluster- III	Cluster- IV	Cluster- V
Days to 50% heading	72.0	65.4	66.2	68.8	65.1
Days to 75% maturity	110.8	107.2	108.2	109.8	106.9
Plant height (cm)	71.5	71.2	81.0	83.8	72.3
Number of tillers per plant	5.6	6.1	6.0	6.8	5.6
Spike length (cm)	7.0	7.3	7.0	8.1	6.2
Number of grains per spike	29.3	38.8	31.1	45.6	27.2
1000-grain weight (g)	34.6	41.4	36.5	41.8	32.5
Grain yield (t ha^−1^)	3.0	3.6	3.4	4.0	2.9
Amylose content (%)	20.2	23.7	23.5	24.6	24.9

#### Principal component analysis (PCA) based on agro-morphological parameters and amylose content in barley genotypes

PCA was performed using quantitative agro-morphological variables for 50 genotypes of barley in order to clarify the patterns of genetic variability. It was found that 99.0% of the total fluctuations were explained by 5 of the main components with an eigenvalue greater than 1.0. The coefficients used to identify the data’s primary components are displayed in Table 9. Table 9 lists the contributions of the quantitative traits to the total genetic diversity. The extent to which each attribute contributes to genetic variety is indicated by its distance from the graph’s center or mean. The correlation between the variables under study and the generated components was ascertained by scaling these coefficients. The specifics are provided below: The first principal component, which determined primarily the genetic variability in the plant height (0.360), number of grains per spike (0.824), and 1000-grain weight (0.411), had a positive high magnitude addition to the PC1, while the days to 50% heading (0.07), days to 75% maturity (0.081), number of tillers per plant (0.050), spike length (0.070), grain yield (0.044), and amylose content (0.007) had a positive low magnitude load on this principal component. Together, these factors accounted for 54.0% of the total variations found in agro-morphological traits. The second main component, which represented the differences in plant height (0.866) with a reasonably positive high magnitude, explained 20% of the overall variance reported in agro-morphological variables. Although to a lesser extent, the days to 50% heading (0.074), days to 75% maturity (0.071), and amylose content (0.013) were all good. Conversely, the weights of the 1000-grain weight (− 0.462), number of tillers per plant (− 0.005), spike length (− 0.003), number of grains per spike (− 0.160), and grain yield (− 0.013) were all negative.

**Table 9:** PCA for agro-morphological traits and amylose content in barley.

Traits	PC1	PC2
Eigen value	9.11	5.50
Cumulative eigen value	9.11	14.61
Total variance (%)	54	20
Cumulative variance (%)	54	74
Traits		
Days to 50% heading	0.07	0.074
Days to 75% maturity	0.081	0.071
Plant height (cm)	0.360	0.866
Number of tillers per plant	0.050	− 0.005
Spike length (cm)	0.070	− 0.003
Number of grains per spike	0.824	− 0.160
1000-grain weight (g)	0.411	− 0.462
Grain yield (t ha^-1^)	0.044	− 0.013
Amylose content (%)	0.007	0.013

### Genetic diversity assessment in barley genotypes based on microsatellite (SSR) markers

Using a total of 20 SSR markers that represent each of the 10 chromosomes, the genetic diversity of 50 genotypes of barley was evaluated. A significant level of heterogeneity among barley genotypes was represented in the amplified profile obtained from a 3% agarose gel (Figs S1 to S2). The number of allelic markers produced by various primer pairings varied (Table 10). Out of the twenty SSR markers, three primer pairs produced four alleles each, thirteen pairs produced three alleles, and four pairs amplified two alleles apiece. After looking at 50 different barley genotypes, 59 different alleles were discovered for each of the 20 primer pairs. The number of alleles produced by GBM1411, GBM1468, Bmag0023, and GBM1102 per locus varied from a minimum of two to a maximum of four, which were amplified by Bmac0213, Bmac0163, and Bmag0135. There were 2.95 alleles on average per locus. With a mean of 0.50, the main allele frequency ranged from 0.32 to 0.74 (Table 10). Bmac0163 was the marker with the lowest major allele frequency (0.32) and the greatest major allele frequency (0.74). With a mean of 0.60, the gene diversity varied from 0.38 to 0.72 (Table 10). Gene diversity was highest (0.72) for Bmac0163 and lowest (0.32) for the marker Bmag0023.

**Table 10:** Summary statistics of the genotyping assay of 50 barley genotypes.

Serial number	Primer	Major allele frequency	Number of alleles	Gene diversity	Heterozygosity	PIC	Allele size range
1	Bmag0211	0.40	3.00	0.66	0.00	0.58	170–180
2	GBM1411	0.52	2.00	0.50	0.08	0.37	145–155
3	Bmac0213	0.50	4.00	0.65	0.00	0.60	160–175
4	GBM1468	0.67	2.00	0.44	0.14	0.34	160–170
5	Bmac0134	0.50	3.00	0.62	0.00	0.54	140–158
6	Bmag0749	0.62	3.00	0.54	0.00	0.48	166–182
7	GBM1280	0.44	3.00	0.65	0.00	0.58	135–145
8	scssr25691	0.44	3.00	0.64	0.00	0.57	178–198
9	Bmag0023	0.74	2.00	0.38	0.08	0.31	137–142
10	scssr20569	0.44	3.00	0.64	0.00	0.56	178–198
11	Hvleu	0.48	3.00	0.62	0.00	0.55	251–266
12	BMAG0375	0.62	3.00	0.54	0.00	0.48	130–145
13	HvLOX	0.54	3.00	0.59	0.00	0.51	150–170
14	Bmac0163	0.32	4.00	0.72	0.00	0.67	138–154
15	HVM65	0.40	3.00	0.65	0.00	0.58	133–143
16	Bmac0018	0.48	3.00	0.63	0.00	0.56	128–148
17	Bmac0040	0.46	3.00	0.64	0.00	0.57	228–246
18	Bmag0135	0.34	4.00	0.72	0.00	0.67	140–160
19	GBM1102	0.56	2.00	0.49	0.12	0.37	170–180
20	EBmac0603	0.44	3.00	0.62	0.00	0.54	145–157
Mean		0.50	2.95	0.60	0.02	0.52	

Among the barley genotypes, the lowest heterozygosity was found (range 0.00–0.12; mean 0.02) (Table 10). Nonetheless, heterozygosity among the 50 barley genotypes was found using certain primers, namely GBM1411 (0.08), GBM1468 (0.14), Bmag0023 (0.08), and GBM1102 (0.12). To evaluate the degree of genetic variety across 50 genotypes, the polymorphism information content value was computed for each of the 20 simple sequence repetitions encompassing all 10 chromosomes. The power of polymorphism among these primer pairs varied greatly, with an average of 0.52 per locus and a range from the minimum level of 0.31 shown by primer pair Bmag0023 on chromosome number 3 to the maximum level of 0.67 reflected by primer pair ‘Bmac0163’ on chromosome number 5 and Bmag0135 on chromosome number 7 (Table 10). There were nine markers with PIC > 0.55.

#### Details of cluster analysis using molecular data

**Fig. 1:**
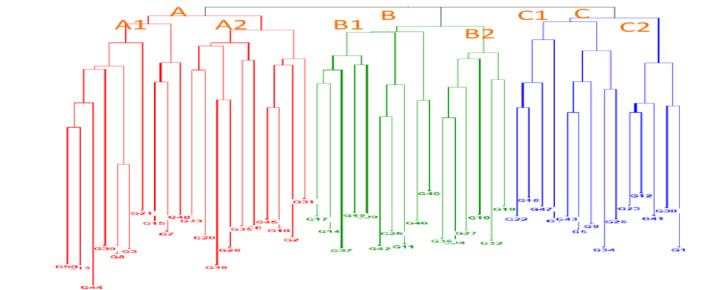
Cluster analysis representing the genetic diversity and genetic connection among barley genotypes by 20 SSR markers. A = cluster, A1 & A2 = subcluster; B = cluster, B1 & B2 = subcluster; C = cluster, C1 & C2 = subcluster.

The dendrogram categorized the 50 genotypes into four major clusters, viz*.,* Cluster-A, B, and C. Cluster-A comprised of 20 genotypes divided in three sub- clusters, i.e., A1 with ten genotypes (G50, G13, G44, G30, G8 and G3, G21, G15, G7, G48, G33), A2 with ten genotypes (G33, G20, G49, G28, G35, G6, G45, G18, G2 and G31). Cluster-B grouped 16 genotypes, which were further split into two sub-clusters -B1 and B2. B1 cluster with ten genotypes (G17, G14, G37, G49, G9, G42, G25, G11, G46, and G40) and six genotypes in cluster B2 (G36, G4, G27, G16, G32, G19). Cluster-C comprised 9 genotypes together, which were further split into two sub-clusters -C1 and -C2, I.e., C1 with five genotypes (G22, G16, G47, G7, G5, G9, G26, G34, and G43) and C2 with five genotypes (G23, G12, G41, G38, and G1). (Fig. 1).

#### Principal coordinate analysis (PCoA)

**Fig. 2:**
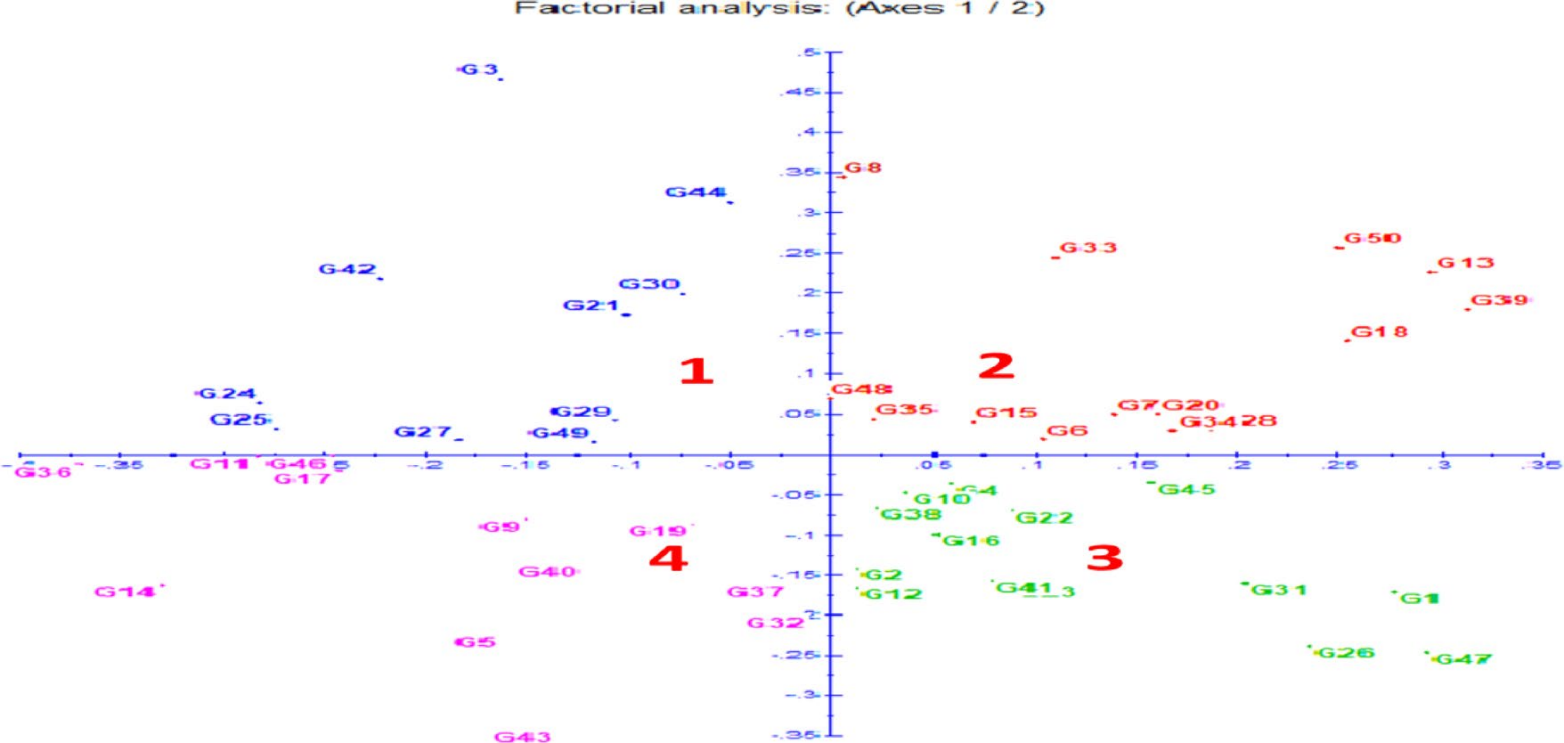
Distribution of 50 barley genotypes based on principal coordinate analysis (PCoA). 1 = quadrangle-1, 2 = quadrangle-2, 3 = quadrangle-3, 4 = quadrangle-4.

Principal coordinate analysis (PCoA) is a tool for exploring and visualizing similarities and differences across genotypes based on a distance matrix, which provides a graphical representation of germplasm diversity. Hence, PCoA was used to reveal the genetic relationships among the 50 genotypes (Fig. 2). Genotypes were found to be uniformly distributed among the four quadrangles in a scatter plot. Barley genotypes were found in four quadrangles. Thirteen genotypes (G1, G3, G8, G11, G21, G24, G25, G27, G29, G30, G42, G44, G48 and G49) were distributed in quadrangles-1; thirteen genotypes (G6, G7, G13, G15, G18, G20, G28, G33, G34, G35, G39, G45 and G50) were in quadrangle-2; thirteen genotypes (G1, G2, G4, G10, G12, G16, G22, G23, G26, G31, G38, G41 and G47) were in quadrangle-3 and eleven genotypes (G5, G9, G14, G17, G19, G32, G36, G37, G40, G43 and G46) were in quadrangle-4.

## Discussion

The exploitation of genetic diversity for crop improvement is vital and depends upon the study of all parameters related to the exploration of genetic diversity^42^. It is obvious that the elucidation of genetic diversity is extremely necessary for the effective maintenance, evaluation and utilization of germplasm because germplasm is the only source to be exploited for the development of new varieties during breeding programs^43^. Breeders inserted desirable genes into the genome of plants and removed the unwanted ones to get favorable breeds^44^. In the current study, agro-morphological, amylose content, and molecular markers were used to assess barley genotypes from ICARDA. Here, the findings from the current study on genetic diversity in barley genotypes are thoroughly examined and contrasted with previous research.

### Genetic diversity assessment in barley genotypes based on agro-morphological traits

Using agro-morphological parameters, we examined genetic diversity and genetic interactions between 50 different genotypes of barley in this study. For every attribute under study, a substantial difference was found between the genotypes. Bedasa et al.^45^ also observed a highly significant variance in the number of tillers per plant, plant height, days to maturity, days to heading, and thousand seed weight. The genotypes displayed a wide range of variance based on plant height. The genotypes between dwarf and tall mature genotypes varied from 67.2 to 90.4 cm. Breeders interested in screening diversity for lodging-tolerant genotypes will benefit from the large range of variation seen in dwarf and tall genotypes. For the most part, a large range of variation was noted for the features. This suggested that there was significant variance among the genotypes that were taken into consideration for the study. Similarly, Addisu and Shumet^46^ found that among the 36 barley landraces they studied, there were significant differences in the following traits: plant height, biomass per plant, number of seeds per spike, spike length, and number of tillers per plant. According to Ferreira et al*.*^38^, barley landraces differ greatly in characteristics such as biological yield, grain production, and days to maturity.

### Genetic diversity assessment in barley genotypes based on amylose content

An effective characteristic of the production and functionality of starch is the apparent amylose and amylopectin ratio^47^. According to studies, enhanced amylose food is responsible for increasing resistance against diseases and it improves health by producing a lower glycemic index^48,49^. The genetic background and the environment both have an impact on the variance and availability of amylose content in cereals^50^. Many genotypes with adequate levels of amylose and amylopectin content are revealed by this inquiry. Maximum genotypes in this investigation resulted in a normal amylose concentration of 20–30%. Reports state that the typical cereal has an amylose content of 18–33% and an amylopectin content of 72–82%. Only two genotypes had more than 85% amylopectin, and we did not find any high amylose content > 30% genotypes. Out of the 50 genotypes, 10 genotypes exhibited amylose contents of more than 27%. It has been established that the gene amo1 is responsible for barley genotypes with amylose levels above 45%^12^. There is clear evidence that higher temperature increases overall amylose content in a cultivar dependent on its genetic background; in addition, environmental factors also affect the amylose content^51,52^. Similar to genetic background and environmental influences, seed size is a significant characteristic that is essential to amylose production^53,54^.

### Correlation of grain yield with other traits

In this investigation, the number of tillers per plant, spike length, plant height, and thousand seed weight all showed a positive and very significant (*p* < 0.01) link with grain yield. The positive and significant correlations of dependent variables with the independent variable indicate the direct increment of the independent variable with the increment of any dependent variable^55–57^. This implied that a considerable rise in barley grain production would result from any improvement in these characteristics. As a result, these characteristics may be used as selection criteria to increase barley yield. Tigist^58^ found a positive and extremely significant correlation between grain yield and biomass yield, thousand kernel weights, and the number of tillers per plant. Al-Tabbal and Al-Fraihat^59^ discovered that there was a strong positive genetic and phenotypic association between the number of kernels per main spike and the grain yield per plant. The number of kernels per main spike and the fertile tiller number, which were significant in connection with grain yield, showed multiple correlations of characters (0.36) that were significantly different from the multiple correlation of all characteristics (0.96). The population’s overall variability for yield improvement, as determined by multiple correlation, was 36%, whereas all other features accounted for 96% of the variability. Fertile tiller quantity and number of kernels per main spike were responsible for 36% of the variability. It was determined that choosing high-yielding barley cultivars primarily depends on the number of fertile tillers and the quantity of kernels per main spike. Ferreira et al*.*^38^ found a positive and highly significant correlation between plant height and days to maturity, which is consistent with this finding. Plant height, the number of tillers per plant, grain yield, 1000-grain weight, and spike length all showed a positive and statistically significant correlation. Fantahun et al*.*^60^ showed a weak but significant connection (r = 0.38***) between grain and biomass yield. A noteworthy, elevated, and adverse correlation coefficient (− 0.72***) was noted between the number of seeds per spike and the 1000 kernel weight. Farmers are drawn to grains with a positive association between biomass and production, since it is supported by the current research as a feed and food crop. With the majority of the enhanced barley gene pool coming from overseas sources, the gap between the local and improved barley gene pools may be reduced by blending the improved materials with farmer-variety barley. According to Bano et al*.*^61^, there was a positive association found between spike length, thousand kernel weight, and grain yield, indicating that farmer varieties were chosen based on the provision of both attributes.

### Cluster analysis and principal component analysis

Five clusters (Cluster I, Cluster II, Cluster III, Cluster IV, and Cluster V) were created from the 50 genotypes used in this investigation. Azam et al*.*^.^^62^ grouped 166 genotypes into seven clusters, and Safath et al*.*^63^ grouped 14 genotypes into four clusters. Similarly , Tahir et al*.*^64^ examined 36 Ethiopian barley varieties and grouped these genotypes into seven groups. Two genotypes each were found in clusters I, III, and IV of their report, whereas cluster IV had eight genotypes. Additionally, they stated that there was significant genetic variation among the genotypes for days to heading, days to maturity, plant height, spike length, number of tillers per plant, number of kernels per spike, biomass yield per hectare, harvest index, and thousand-grain weight, as evidenced by the distribution pattern of the genotypes into different clusters. Cluster-V is home to early-maturing lines, while Cluster-I is home to late-maturing lines. The genotypes with the highest yields were found to be grouped in cluster IV and cluster V, which had the lowest grain yield cluster mean. In accordance with these findings, Bedasa et al*.*^45^ examined 102 more Ethiopian barley landrace accessions and categorized the genotypes into five groups. Cluster I had the highest number of genotypes (44), whereas Cluster V had the lowest number of genotypes (two).

The current study found that while trait contribution varied among the three PCs, phenology features and the amount of grains per spike with the highest load had the greatest impact on PC 1. Other agronomic variables, such as plant height, thousand kernel weight, grain yield, and peduncle length, had more contributions in the positive direction. Karaman et al*.*^65^ investigated barley varieties using heatmap and PCA to identify superior ones. They found that 1000-grain weight was associated with the heading time, whereas starch ratio was associated with the grain yield. According to Dido et al*.*^66^, the first PC was significantly loaded by days to going in the negative direction. PC2 showed a negative correlation with grain yield, 1000-grain weight, and the number of seeds per spike, but a positive correlation with plant height, days to heading, days to maturity, and amylose content. The results of Gadissa et al*.*^67^ showed that the single leaf area, flag leaf length, and leaf width of the second PC had high factor loading. PC3 was found to be highly influenced by grain filling time and grain yield, but negatively impacted by other phenologic and agronomic characteristics such as plant height, spike length, and number of seeds per spike. Traits with higher loading in the first PCs contribute a higher share of the phenotypic variance among the lines under review in this research, as PC1 accounts for a larger portion of the variability.

### Genetic diversity assessment in barley genotypes based on microsatellite (SSR) markers

Using SSR markers, we examined the genetic diversity and interactions between 50 different barley genotypes in the current study. Because they might have shared genetic material through breeding programs, genotypes with the same origin are thought to imply restricted genetic diversity, according to Hamza et al*.*^68^. The mean PIC value in this experiment was 0.60, indicating that these SSR marker sets were positively informative. One criterion for evaluating the markers of differentiation power is the Polymorphism Information Content or PIC. The number of alleles and their relative frequency determine a marker’s discriminating capacity. When PIC and allele count were compared, it was found that markers with a high allele count also had a high PIC rate, making them useful for population studies. The highest frequency of major alleles was shown to be associated with markers with fewer alleles, according to a comparison of major allele counts. As a result, differentiation power was greater at a location with a lower major allele frequency. Increasing the number of alleles generally increases PIC and genetic diversity. Current Study detected three alleles ranging from 166 to 182 bp, suggesting higher polymorphism and genetic variability among the barley genotypes analyzed, whereas Elakhdar et al*.*^35^ reported only a single allele of 166 bp for Bmag0749, which may indicate a narrower genetic base in their sample set. This result implies a strong and direct correlation between these two variables. The examined lines were arranged into four groups, and a dendrogram was created using UPGMA techniques to show the relationships between the lines. The barley genotypes clustered partially based on origin geography, as seen by the dendrogram.

In conclusion, a significant amount of genetic diversity was documented in the genotypes under investigation in terms of agro-morphological characteristics. Among the barley genotypes, the primary agronomical factors that contributed to the overall genetic diversity were grain yield (t ha^-1^), plant height, number of grains per spike, and 1000-grain weight.

There was a large variation in the ratios of amylose to amylopectin across the various barley genotypes. The current findings indicated that the amylose and amylopectin content ranges and averages were lower than anticipated given the species’ ploidy. Grain yield was positively and significantly correlated with plant height, number of tillers per plant, spike length, number of grains per spike, and 1000-grain weight. Linked genes exist as a correlation between different features, which play a significant role in choosing the best trait for breeding. The genotypes G20, G7, G18, G28, G41, G45, G50, G13, and G39 showed a high yield and can be selected based on their notable performance for future breeding. The highest amylose content was obtained from G4, G5, G8, G12, G19, G34, G6 G37, and G41. These genotypes could be selected for quality improvement for preferred amylose and amylopectin content. Based on agro-morphological traits and amylose content, 50 genotypes were grouped into five genetically distinct clusters. The clustering analysis gives an idea for developing a diverse genetic pool for a successful breeding program. The more diversified genotypes can be employed as parents for extensive hybridization. Using twenty SSR markers a higher allelic diversity in the genotypes was observed to offer significant genetic resolution. We have to choose parents from different cluster groups. The majority of the markers utilized in this investigation might be used in association and linkage studies because of the observed allelic variety.

## Supplementary Information

Below is the link to the electronic supplementary material.

Supplementary Material 1

## Data Availability

The data generated or analyzed during the current study are provided within the manuscript and supplementary file.
