# Supplementary figures and images for: Genetic variation of barley genotypes using morphological traits, amylose content, and molecular markers
Source: Sci Rep. 2025 Oct 7;15:34840. doi: 10.1038/s41598-025-19242-w (PMC12504752; doi:10.1038/s41598-025-19242-w)

## Slide 1
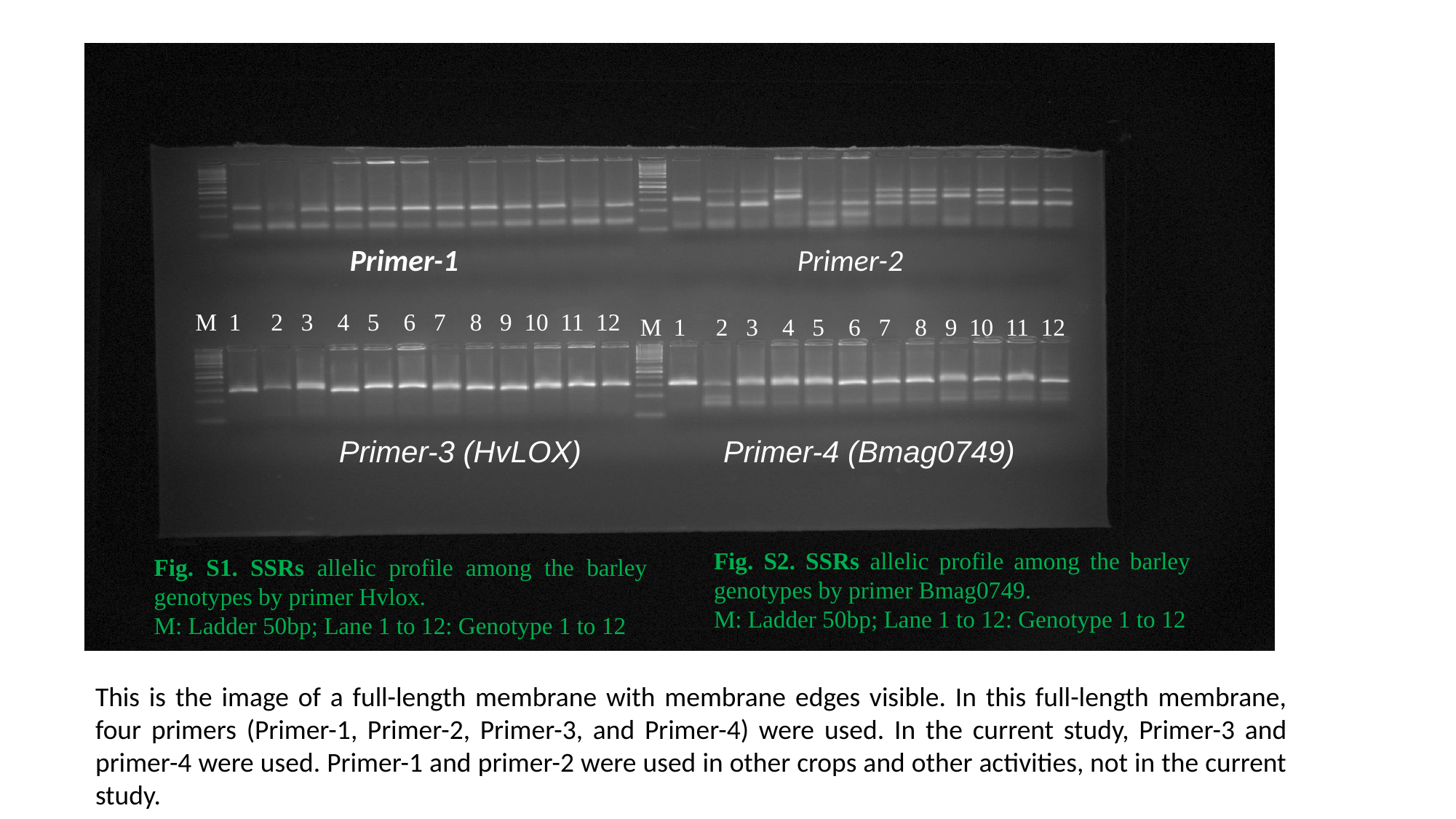

## Slide 2
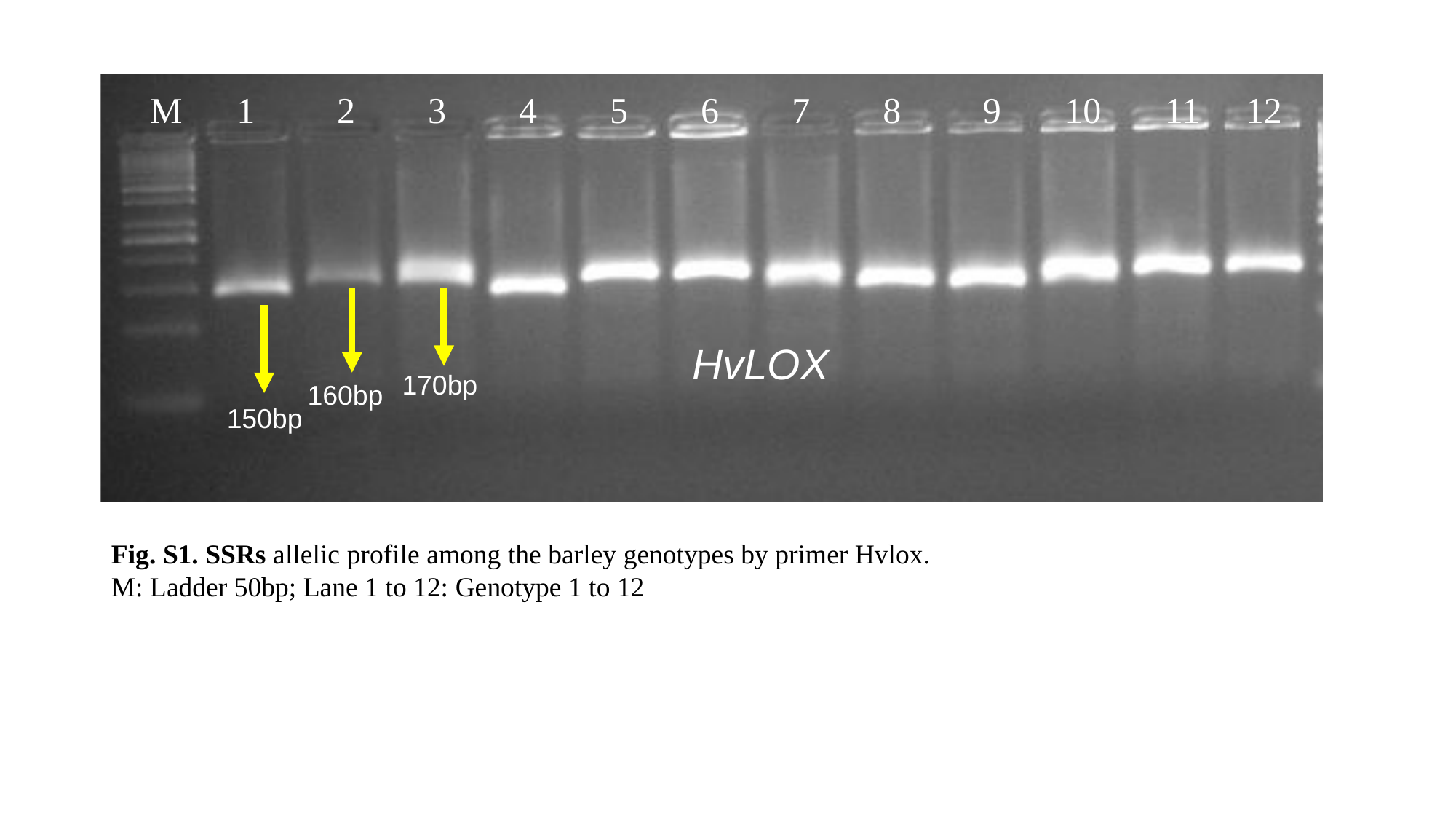

## Slide 3
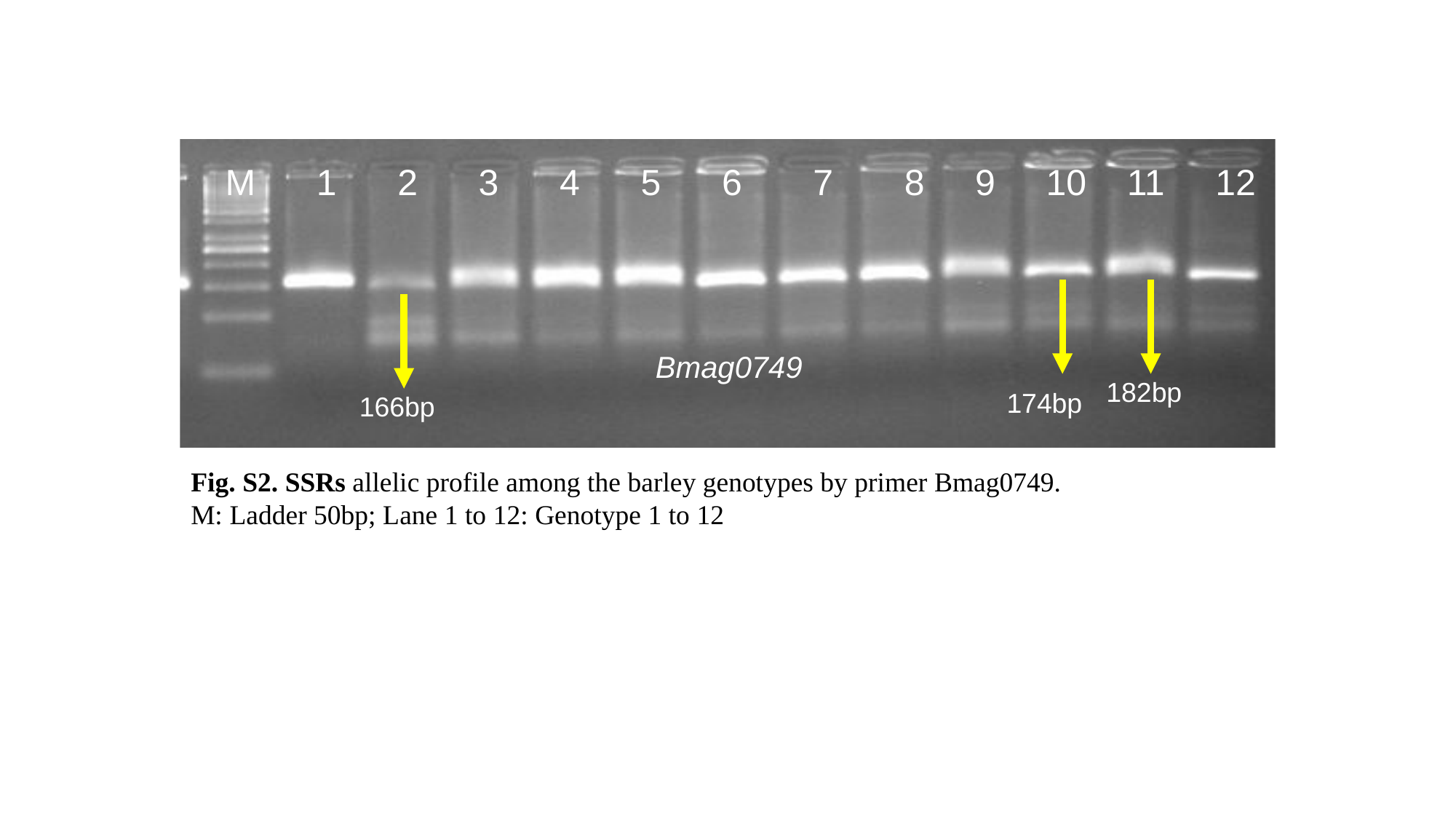

Supplement: Supplementary file 1 — Supplementary Material 1 [file 41598_2025_19242_MOESM1_ESM.pptx]
